# The associations and roles of microRNA single-nucleotide polymorphisms in cervical cancer

**DOI:** 10.7150/ijms.57990

**Published:** 2021-04-07

**Authors:** Yaheng Li, Chuanyin Li, Shuyuan Liu, Jia Yang, Li Shi, Yufeng Yao

**Affiliations:** Department of Immunogenetics, Institute of Medical Biology, Chinese Academy of Medical Sciences & Peking Union Medical College, Kunming 650118, Yunnan, China.

**Keywords:** cervical cancer, miRNAs, single nucleotide polymorphisms, regulation

## Abstract

Cervical cancer is one of the fourth most common gynecological malignancies and has been identified as the fourth leading cause of cancer death in women worldwide. MicroRNAs (miRNAs) are single-stranded sequences of noncoding RNAs that are approximately 22-24 nucleotides in length. They modulate posttranscriptional mRNA expression and play critical roles in cervical cancer. Single nucleotide polymorphisms (SNPs) in miRNA genes may alter miRNA expression and maturation and have been associated with various cancers. This review mainly focuses on the roles of SNPs in miRNA genes in the development of cervical cancer and summarizes the research progress of miRNA SNPs in cervical cancer and their molecular regulation mechanisms.

## Introduction

### Cervical cancer

Cervical cancer is the fourth most diagnosed common cancer and the fourth death cause of cancer in women worldwide, which makes it a serious threat to women's life and health, especially in developing countries 1. The occurrence and development of cervical cancer can be divided into two main stages: cervical intraepithelial neoplasia (CIN) and cervical cancer 2. Studies have found that cervical cancer is a disease caused by a long-term interaction of multiple factors. Persistent infection with high-risk human papillomavirus (HPV) is a necessary condition for the occurrence of cervical cancer, whereas HPV infection alone is not sufficient to induce malignant transformation. Moreover, host genetic factors also play important roles in the occurrence and development of cervical cancer 3. The persistent infection with high-risk HPV will cause HPV-DNA to integrate into the host genome, leading to increased expression of viral oncogenes E6 and E7 in cervical inflammation which is the cause of cervical cancer, at the same time, inflammation may also lead to abnormal expression of miRNA 4. Shen *et al*. reported that miRNA could affect the replication of high risk HPV DNA, which may influence the life cycle of HPV and the mechanism of HPV-induced tumorigenesis 5. Therefore, the miRNA may play a key role in high risk HPV tumorigenesis. Ellwanger *et al*. reported that the SNPs in miRNA might modulate immune response and viral restriction, cell cycle, proliferation and apoptosis, which may relate to HPV infection 6. In addition, HPV's E6 and E7 oncoproteins are able to induce overexpression of DNA methyltransferase, which can cause abnormal methylation of miRNA genes and be associated with cervical cancer. Therefore, the interaction between the miRNAs and HPV genes may affect the occurrence and development of cervical cancer 4. In recent years, studies on the role of host genetic factors in the development of cervical cancer, especially host miRNAs and their polymorphisms, have been research hot spots worldwide.

### MiRNA

MiRNAs are a class of small, noncoding single-stranded RNA molecules that are encoded by endogenous genes; they are approximately 20-24 nucleotides in length and function as gene regulators in eukaryotes 7. Mature miRNAs regulate the expression of target genes by binding to the 3'-UTR of the target gene through sequence complementation 8 and are involved in various cell biological processes, including cell proliferation, apoptosis, and migration 9. MiRNAs encoding genes are transcribed to long primary transcripts (pri-miRNAs) by RNA polymerase in the cell nucleus. The pri-miRNAs are approximately 300 to 1000 nucleotides in length and are then cleaved to form miRNA precursors (pre-miRNAs) under the action of the Drosha enzyme 10. Pre-miRNAs have stem-loop structures that are approximately 60-70 nucleotides in length, and they are transported from the nucleus to the cytoplasm by exportin-5 and processed into approximately 22-nt mature miRNA duplexes via cleavage by the Dicer enzyme 11. Finally, mature and functional single-stranded miRNA molecules are processed and generated by helicase 12.

### Single nucleotide polymorphisms (SNPs) in miRNAs

Single nucleotide polymorphisms are the most common type of human heritable variation and are caused by the mutation of a single nucleotide at the genome level in every 100-300 base pairs 13. SNPs may occur in coding gene sequences or in noncoding regions, and approximately 90% of functional SNPs are located in noncoding regions, including promoter regions, enhancer regions and noncoding RNA sequences 14. SNPs in miRNA genes, including pri-miRNAs, pre-miRNAs and mature miRNAs, may affect miRNA shearing, processing, and maturation processes; they play important roles in the expression level of mature miRNAs and the combination of miRNAs and their target genes and influence the development of diseases 15-17. Many studies have reported that SNPs in miRNAs are related to different diseases, especially cancers 17-21. SNPs in miRNA genes may affect the miRNA regulation of signaling pathways by affecting the expression of mature miRNAs or the recognition of target genes by miRNAs, thereby playing a role in the occurrence and development of diseases.

## The roles of miRNAs and their SNPs in cervical cancer

A large number of miRNAs with abnormal expression in cervical cancer tissues and cells have been reported recently 22. The abnormal expression of miRNAs in cervical cancer can affect its pathogenesis by affecting downstream signaling molecules and further affect the progression and prognosis of cervical cancer or may be related to the sensitivity of patients to radiotherapy and chemotherapy 23. MiRNAs can affect the treatment effect and survival rate of patients and provide a reliable method for the diagnosis and effective treatment of cervical cancer 24.

These miRNAs with abnormal expression affect the expression of target genes in tumor-related signaling pathways and thus affect the regulation of signaling pathways and ultimately play a pivotal role in the occurrence and development of cervical cancer. For example, miRNAs may function as oncogenes or tumor suppressors to regulate the occurrence and development of cervical cancer through special signaling pathways, including the PI3K-AKT pathway 25, Notch pathway 26, E6-p53 pathway 27, E7-pRb pathway 28, Wnt/β-catenin pathway 29, NF-κB pathway 30, and Hedgehog pathway 31.

Studies have reported that SNPs in pri-miRNA and pre-miRNA genes may interfere with the maturation process or degradation of miRNAs by destroying the secondary structure, reducing the number of mature miRNAs and affecting the expression of mature miRNAs 32, 33. The SNP site located in the mature miRNA may lose its combination with the mRNA target sequence or change its binding efficiency to the target sequence due to the mutation of the base site 34. Therefore, SNPs in miRNA genes may affect the regulation of miRNAs on related signal pathways and the biological functions of cells in various ways by affecting the expression of mature miRNAs or the recognition of target genes by miRNAs 6, 35, 36. Moreover, some miRNAs have targeting relationship with multiple genes, at the same time; a certain target gene could also bind to multiple miRNAs 37. The regulation of complex miRNAs may have the ultimate biological effect, thereby playing a role in the occurrence and development of different kinds of diseases including cervical cancer (Table 1).

### SNPs in pri-miRNAs and cervical cancer

MiR-34 is considered to be the first miRNA that is directly regulated by p53, and it has been reported to be dysregulated in many types of cancer 38, 39. The miR-34 family consists of the 5p and 3p strands of miR-34a, miR-34b and miR-34c 40. Yuan *et al*. reported that the SNP rs4938723 in the promoter of pri-miR-34b/c was significantly associated with cervical cancer, and the C allele was a risk factor for cervical cancer in a Chinese Han population. In addition, the CT genotype was significantly associated with the clinical staging and poor prognosis of patients 41. Previous studies have shown that miR-34 family members can directly target p53, thereby inducing cell apoptosis, DNA repair, angiogenesis and other biological processes 27. At the same time, p53 can regulate miR34 family members, thereby inhibiting the expression of sirtuin 1 to increase p53 activity and form a p53-miR-34 positive feedback loop 42. Their study found that the CT and CC genotypes of rs4938723 combined with the CG and CC genotypes of TP53 Arg72Pro increased the risk of cervical cancer by 2.21 times. Studies have showed that the expression of miR-34a was downregulated in cervical cancer tissues 43 and miR-34a can bind to the 3'-UTR of Notch1 and Jagged1 and inhibit the invasiveness of cervical cancer cells by regulating the Notch pathway and its downstream matrix-degrading enzymes 26. Therefore the two genotypes in rs4938723 and TP53 together may affect the expression of miR-34b/c and p53 and ultimately increase the risk of cervical cancer.

MiR-218 is encoded by the intron of the SLIT2 tumor suppressor gene 44, and its expression level is reduced in breast cancer 45, lung cancer 46, and gastric cancer 47. Similar to that in other cancers, the expression level of miR-218 in cervical cancer cells and tissues is significantly lower than that in paracancerous tissues and normal tissues and can inhibit the proliferation, migration and invasion of cervical cancer cells 47. Several studies have reported that the rs11134527 SNP in the intron of pri-miR-218 is related to hepatocellular carcinoma 48, gastric cancer 49 and breast cancer 50. Research of Zhou and Shi *et al*. found that the SNP rs11134527 in miR-218 was associated with cervical cancer in a Chinese Han population, and the GG genotype can reduce the risk of cervical cancer 51, 52. Predicting the folding of RNA by RNA-fold found that the mRNA structure of rs11134527 allele A to G has changed significantly. These findings further indicate that the SNP site may cause changes in the expression of miR-218 and affect the process of miRNA binding, and therefore is related to the susceptibility of cervical cancer 52. Li *et al*. showed that the SNP rs11134527 was associated with the development of CIN in cervical cancer in a Chinese Han population, and the A allele of rs11134527 was associated with a lower risk of CIN 53. Therefore, the SNP site located in pri-miR-218 mRNA may change its local secondary structure, resulting in changes in the expression of miR-218 and affecting the binding process of miRNA; therefore, this SNP may be related to susceptibility to cervical cancer.

### SNPs in pre-miRNAs and cervical cancer

Previous study found that miR-27a was highly expressed in cervical cancer tissues and in HeLa and C33A cells 54. Xiong *et al*.'s study found that the SNP rs895819 located in the promoter of pre-miR-27a has a significant association with the risk of cervical cancer in a southern Chinese Han population 55. The T allele and CT and TT genotypes have a significant risk reduction compared with the C allele and CC genotype. Compared with the CC genotype, the TC and TT genotypes were significantly associated with a lower risk of cervical cancer in the recessive inheritance model. However, Subsequently Chen *et al*. reported that the SNP rs895819 was associated with cervical cancer in a dominant genetic model in a southern Chinese Han population 56. Compared with the TT genotype, the TC and CC genotypes significantly reduce the risk of cervical cancer in a dominant inheritance model. The discrepancy between the studies of Xiong and Chen could be caused by different sample sizes and baseline characteristics 57. Sun *et al*. reported that overexpression of miR-27a upregulates the expression of B4GALT3, promotes carcinogenic activity through the β1-integrin pathway, and may be a potential biomarker for cervical cancer 54. Another similar study on miR-27a also showed that miR-27a was highly expressed in cervical cancer tissues, promoted the expression of the target gene inositol polyphosphate-1-phosphatase (INPP1), and promoted tumorigenic activity 58. Much more research is needed to detect the potential related mechanisms and functions between rs895819 in miR-27a and cervical cancer in the future.

Mature miR-124 is processed by pre-miR-124-1, pre-miR-124-2 and pre-miR-124-3, all of which have reduced expression in a variety of tumor tissue types 59. Xiong *et al*. reported that the G allele of the SNP rs531564 in the promoter of miR-124-1 was a protective factor in cervical cancer in a Chinese Han population 60. Compared with individuals with the CC genotype, individuals with the CG and GG genotypes have a significantly lower risk of developing cervical cancer 60. Subsequently, Li *et al*. showed that the C allele of rs531564 was the risk allele of cervical cancer in a Chinese Han population, and this SNP was related to the progression from CIN to cervical cancer 53. Their research suggests that SNPs in miRNA genes may affect the expression or function of miRNAs 61. Therefore, the SNP in miR-124 may be related to the expression of miR-124 and further related to cervical cancer. Study of Wan *et al*. found that miR-124 can inhibit the mimicry and cell movement of angiogenesis by targeting amotL1 62. Moreover, research of Shen *et al*. found that miR-124 negatively regulates the target lncRNA NEAT1 and affects the migration and invasion of HeLa cells, EMT and activation of the NF-κB pathway 30.

Several studies have found that miR-126 is usually underexpressed in human colorectal cancer 63, breast cancer 64 and cervical cancer 65. Yan *et al*. reported that rs4636297 in miR-126, which was located 12 bp downstream of the pre-miR-126 sequence, was associated with CIN and cervical cancer in a Chinese Han population 66. The results of the study by Yan *et al*. indicated that the T allele confers a higher risk of developing CIN and cervical cancer 66. The association of the SNP rs4636297 with cervical cancer may be because the SNP is related to Drosha's recognition and cleavage of pri-miRNA 34. Therefore, the SNP rs4636297 may affect the expression of miR-126, which affects the occurrence and development of cervical cancer. MiR-126 can inhibit the expression of MMP2 by targeting ZEB1, which inhibits the protein expression of p-JAK2 and p-STAT3 and inactivates the JAK2/STAT3 signaling pathway, which inhibits the proliferation, migration and invasion of cervical cancer cells 65. Therefore, it will be valuable to carry out functional and associational researches to study the roles of rs4636297 in human cancers in the future for the reason that the location of this SNP might affect biogenesis.

MiR-137 is underexpressed in cervical cancer tissues and cell lines and can inhibit the development of tumors 67. Chen *et al*. reported that the SNP rs1625579 in the intron of pre-miR-137 was associated with a significant reduction in the risk of cervical cancer in a Sourthern Chinese population. Compared with the TT genotype, the TG and GG genotypes had a better protective effect on cervical cancer patients 56. They showed that the polymorphism of miR-137 rs1625579 may be positively correlated with the expression of miR-137, which in turn affects the occurrence of cervical cancer. More confirmation researches are required. Overexpression of miR-137 in cervical cancer cells can regulate the expression of downstream target genes (EZH2^67^ or GEEM1^68^) to regulate signaling pathways such as TGF-β/Smad to inhibit cell proliferation and migration.

Lui *et al*. detected the expression of miRNAs in six types of cervical cancer cell lines and normal cervical samples, and they found that cancer samples can repeatedly show increased miR-21 expression 69. Zhang *et al*. reported that there were significant differences in the alleles and genotypes of rs1292037, which is located 210 bps from the 3' end of pre-miR-21, in cervical cancer patients in the sensitive and resistant groups. The rs1292037 locus with the G allele in the miR-21 gene may increase the resistance of cervical cancer patients to cisplatin plus paclitaxel l in a Northern Chinese population 70. Yao *et al*. confirmed that miR-21 was significantly overexpressed in human cervical squamous cell carcinoma tissues and cell lines and found that miR-21 regulates the proliferation, apoptosis and migration of HPV16-positive cervical squamous cells by regulating the expression of the target gene CCL20^71^. They also showed that the overexpression of miR-21 was related to lymph node metastasis and advanced disease 71. Xu *et al*. reported that the overexpression of miR-21 inhibited the expression of the mRNA of the target gene PTEN in cervical cancer cell lines to promote the proliferation, migration and invasion of cervical cancer cells 72. Du *et al*. detected the sensitivity of cervical cancer cells to paclitaxel and found that the inhibited expression of miR-21 can inhibit cell proliferation and colony formation by regulating the PTEN/AKT pathway and improve the PTX sensitivity of cervical cancer cells 25. More research is needed to analyze the molecular regulation mechanism of rs1292037 on the transcription level and methylation level of miR-21 and its role in cervical cancer progression and chemoresistance, which may indicate its use in the treatment cervical cancer.

The expression of miR-149 is upregulated in cervical cancer tissues and cells 33. A large number of studies have shown that the rs2292832 in the promoter of pre-miR-149 is related to a variety of cancers, including breast cancer 73 and lung cancer 74. Wang *et al*. reported that for the polymorphic site rs2292832 of miR-149 in cervical cancer tissues and HeLa and SiHa cells, the TC and CC genotypes increased the risk of cervical cancer compared with the TT genotype 75. We speculate that rs2292832 may affect the expression of miRNA by affecting the stability of the secondary structure of miR-149 and may further affect the regulation of miR-149 to target genes, thereby playing a role in the occurrence and development of cervical cancer.

The reason behind the association of SNPs with cervical cancer could be because SNPs located in pre-miRNAs may affect the expression of mature miRNAs and be involved in the binding of certain nuclear factors to miRNAs during miRNA processing.

### SNPs in mature miRNAs and cervical cancer

MiR-146a is abnormally expressed in a variety of cancers 76-79. Upregulating the expression of miR-146a may affect the proliferation of cancer cells 80. The SNP rs2910164 is located in the 3p chain of mature miR-146a. Its polymorphic sites involve mismatches in the hairpin of pre-miR-146a, which leads to changes in its processing and reduces the expression of mature sequences 81, thereby affecting the expression of downstream signaling molecules. Hu *et al*. found that the expression of miR-146a was upregulated in HeLa cervical cancer cells and C33A cells, and the rs2910164 SNP increased the amount of mature miRNAs in cervical cancer cells. Similarly, Li *et al*. reported that miR-146a can act as an oncogene, affect the expression of the target gene TRAF6 and regulate the differentiation of Th17 cells through NF-κB signaling to regulate the growth and apoptosis of cervical cancer cells 82, 83. The expression of miR-146a can downregulate the expression of receptor-related kinase 1 (IRAK1) and TNF receptor-related factor 6 (TRAF6), promote the expression of cyclin D1, and promote the occurrence of cervical cancer. It was found that after recombinant plasmid transfection, the expression of miR-146a in the C allele was higher than that in the G allele in cervical cancer cells, while the expression of miR-146a in the C allele in CRL-2615 cells was lower than that in the G allele. However, this SNP played the opposite role in immortalized nontumorigenic endocervical CRL-2615 cells 82. Therefore, the role of this SNP site varied in different cervical cell lines.

Previous studies reported that the expression of miR-196a in human cervical cancer tissues and cell lines was significantly upregulated 84. Many studies have found that the SNP rs11614913 located in mature miR-196a2 was associated with a variety of human cancers, including breast cancer 85, non-small cell lung cancer 86, ovarian cancer 87 and hepatocellular carcinoma 88. Study of Nisha *et al* showed that the T allele in the SNP rs11614913 was associated with a reduced risk of cervical cancer development in an Indian population 89. Similarly, the research results of Yan *et al*. in a Chinese Han population were consistent with those of Nisha *et al*. 66. The SNP rs11614913, which is located in the mature sequence of miR-196a2, may affect the biological function of miRNA and the recognition of target mRNA, thereby affecting the occurrence and progression of cervical cancer 33. The key role of miR-196a was in regulating cell cycle checkpoints and cervical cancer cell proliferation. Moreover, they confirmed the correlation between the proliferation of cervical cancer cells was mediated by miR-196a and the downregulation of the target gene FOXO1 p27Kip1 in the PI3K signaling pathway 84.

Therefore, the abnormal expression of miRNAs may directly or indirectly affect the expression of many target genes, and the expression levels of these target genes will also dynamically change under various cell and environmental stimulations. Combining these changes with SNP-related miRNA-level regulation can provide important internal mechanisms for disease development.

## Conclusions and Future perspectives

The occurrence of tumors is the result of the abnormal regulation of signaling pathways related to cell proliferation, differentiation, apoptosis, and the cell cycle. Mutations in the genes that affect different cell signaling pathways could play important roles in the mechanisms of the occurrence and development of cervical cancer. Many studies have found that mutations in miRNA genes may affect the combination of mature miRNAs and downstream target genes by affecting miRNA expression, thereby affecting the regulation of signaling pathways and playing an important role in the occurrence and development of cervical cancer. This review summarizes the SNP sites of miRNAs related to cervical cancer, lays a foundation for in-depth research on the pathogenesis of cervical cancer and identifies new targets for the diagnosis and treatment of cervical cancer.

## Figures and Tables

**Table 1:** SNPs in microRNA genes involved in cervical cancer

SNPs	MicroRNAs	Polymorphism	Consequence	Association in Cervical cancer	Populations [Case(n)/Control(n)]
rs4938723	miR-34b/c	T>C	BTG4: Intron Variant; miR-34b/c: 2KB Upstream Variant	C allele associated with Increased risk	Chinese (328/568) 41
rs11134527	miR-218	A>G	SLIT3: Intron Variant; miR-218-2: 2KB Upstream Variant	G allele associated with decreased risk 51, 52; A allele associated with decreased risk 53	Chinese (703/713) 51 Chinese (1584/1394) 52 Chinese (609/583) 53
rs895819	miR-27a	T>C	miR-27a: Non Coding Transcript Variant	T allele associated with decreased risk 55; C allele associated with decreased risk 56	Chinese (103/417) 55 Chinese (290/445) 56
rs531564	miR-124	G>C	LINC0059: Non Coding Transcript Variant; miR-124-1: 500B Downstream Variant	G allele associated with decreased risk	Chinese (107/208) 60 Chinese (609/583) 53
rs4636297	miR-126	C>T	EGFL7: Intron Variant miR-126: 500B Downstream Variant	T allele associated with increased risk	Chinese (547/567) 66
rs1625579	miR-137	G>T	miR-137HG: Intron Variant	G allele associated with decreased risk	Chinese (290/445) 56
rs1292037	miR-21	A>G	VMP1: 3 Prime UTR Variant; miR-21: 500B Downstream Variant	G allele associated with increased risk	Chinese (165) 70
rs2292832	miR-149	T>C	miR-149: Non Coding Transcript Variant; GPC1: Intron Variant; LOC100130449: Intron Variant	C allele associated with increased risk	Chinese (954/1339) 75
rs2910164	miR-146a	G>C	miR-146a: Non Coding Transcript Variant; miR-3142HG: Non Coding Transcript Variant	C allele associated with increased risk	Chinese 82
rs11614913	miR-196a2	T>C	miR-196a2: Non Coding Transcript Variant	T allele associated with decreased risk	Indian (150/150) 89 Chinese (547/567) 66
